# What Is Worse: A Comparison of Solitary Versus Multifocal Pyogenic Spondylodiscitis Using a Nationwide Analysis of Readmission Rates and Risk Factors

**DOI:** 10.3390/jcm14165784

**Published:** 2025-08-15

**Authors:** Julius Gerstmeyer, Anna Gorbacheva, Clifford Pierre, Mark Kraemer, Colin Gold, Cameron Hogsett, Nick Minissale, Alexander von Glinski, Tobias L. Schulte, Thomas A. Schildhauer, Amir Abdul-Jabbar, Rod J. Oskouian, Jens R. Chapman

**Affiliations:** 1 Swedish Neuroscience Institute, Swedish Medical Center, 550 17th Avenue, Suite 500, Seattle, WA 98122, USA; 2 Department of General and Trauma Surgery, BG University Hospital Bergmannsheil, Ruhr University Bochum, Bürkle-de-la-Camp-Platz 1, 44789 Bochum, Germany; 3 Department of Orthopedics and Trauma Surgery, St. Josef Hospital Bochum, Gudrunstraße 56, 44791 Bochum, Germany; 4 Seattle Science Foundation, 550 17th Avenue, Suite 600, Seattle, WA 98122, USA

**Keywords:** spondylodiscitis, readmission rate, multifocal, spine infection, risk factor

## Abstract

**Background:** Spondylodiscitis is a growing infectious condition with significant morbidity. The impact of multifocal involvement remains understudied. This study compared 90-day all-cause readmission rates between patients with solitary versus multifocal spondylodiscitis and identified the associated risk factors. **Methods:** A retrospective analysis of the 2020 Nationwide Readmissions Database was conducted. Adult patients with primary spondylodiscitis were identified using ICD-10 codes and categorized into solitary or multifocal involvement groups. Demographic, clinical, and surgical data were extracted. Descriptive statistics and multivariate logistic regression were performed. **Results:** Of 6132 patients, 585 (9.6%) had multifocal disease. Multifocal patients were slightly younger (58.9 vs. 60.3 years; *p* = 0.049); had longer hospital stays (14.7 vs. 11.4 days; *p* < 0.001), time to readmission (*p* < 0.001); and surgery was more common (*p* = 0.003). Ninety-day readmission rates were similar (35.6% vs. 34.9%; *p* = 0.766). Type 2 diabetes was the only comorbidity significantly associated with multifocal disease (*p* = 0.020) and independently predicted readmission (aOR 1.236). Surgery and longer length of stay were protective (aOR 0.743; 0.0990). **Conclusions:** Multifocal spondylodiscitis is relatively common but not an independent risk factor for readmission. Readmission rates of both cohorts were similar. Surgery and prolonged hospitalization may reduce readmission risk.

## 1. Introduction

Pyogenic spondylodiscitis (SD) represents a serious challenge to all involved in its management from its onset onwards, with its non-specific clinical symptoms of back pain and constitutional symptoms of mild fever and malaise with or without neurological deficits that may mimic other spinal pathologies [1,2]. Recognized associated chronic medical conditions include common conditions such as diabetes mellitus; liver cirrhosis; use of hemodialysis; malignancy; obesity; use of intravenous drugs; immune system-modifying agents such as systemic steroids and similar biologic agents; and even non-modifiable risk factors such as advanced age and gender [3,4]. There has been a well-recognized global rise in the incidence of pyogenic SD, such as in the case reported in England over a ten-year period, affecting patients aged 70–74 and 75–79, of 117% and 133%, respectively. This increase may be elucidated by an increased availability of modern imaging techniques and an aging and more multimorbid population. Pyogenic SD, predominantly caused by *Staphylococcus aureus*, remains dominant in the Western world. However, in developing countries granulomatous SD—including tuberculous, brucellar, fungal and aspergillar—remain most frequent [5].

In recent years efforts have been made to formally standardize diagnosis, treatment, and complications such as resulting spinal instability [6,7,8]. However, to date, there have been few publications addressing multifocal spondylodiscitis, defined as occurring in more than one anatomical region. Published prevalences of multifocal involvement, in pyogenic or tuberculosis SD, range from a low of 0.9% to a high of 32%, potentially affected by a pathogen-specific variability [9,10,11,12]. A knowledge gap for this disease entity is a lack of exploration of the associated disease burden and the specific disease severity associated with multifocal spinal infections [9]. Currently, there are relatively small studies that have investigated the risk of developing multifocal disease in spondylodiscitis [13,14,15]. As rates of spondylodiscitis have inevitably risen in the setting of an increasingly aging and comorbid general population, a more in-depth exploration of the associated disease burden, as well as morbidity and mortality of patients with multifocal spondylodiscitis, would seem beneficial. The primary objective of this study was to assess the 90-day all-cause readmission rate in patients suffering from solitary spondylodiscitis compared to patients with multifocal disease manifestations, with the secondary objective being an investigation of the effects of comorbidities and treatment on the readmission rates in these patients.

## 2. Materials and Methods

### 2.1. Study Design and Data Source

We conducted a retrospective cohort study utilizing data from the 2020 Nationwide Readmissions Database (NRD), Healthcare Cost and Utilization Project (HCUP), provided by the Agency for Healthcare Research and Quality. Demographic details and hospitalization parameters, including the length of hospitalization, comorbidities, and surgical treatment, were extracted. Comorbidities were defined using ICD-10 codes and primary clinical classifications software refined (CCSR) for International Classification of Diseases (ICD)-10 category codes, Version 2023.1. present at discharge were extracted for analysis [16]. Surgical treatment was identified using a clinical classifications software refined (CCSR) for International Classification of Diseases (ICD)-10-PCS procedures category codes, Version 2023.1. Codes included spine fusion (MST013), discectomy (MST016), corpectomy (MST-18), spinal cord decompression (CNS008), cervicothoracic nerve decompression (PNS002), and lumbosacral nerve decompression (PNS001) [16]. As a measurement of comorbidity burden, the Elixhauser Comorbidity Index for in-hospital mortality and all-cause 90-day readmission was calculated for each patient [17]. Consistent with HCUP guidelines, variables resulting in patient counts of ten or fewer were omitted from all tabulated results to maintain patient confidentiality. Due to the public nature of the data, Institutional Review Board (IRB) approval was not required.

### 2.2. Population

Patients aged over 18 years, diagnosed with pyogenic spondylodiscitis (ICD-10 codes M46.2x, M46.3x, M46.4x according to the ICD-10, Version 2023.1), were included. The exclusion criteria consisted of patients with a history of primary spine surgery, tuberculosis infection, concurrent malignancies, traumatic injuries, or incomplete records. To reliably calculate 90-day readmission rates, only patients discharged within the initial nine-month period of 2020 were evaluated.

### 2.3. Cohorts

The patients were stratified based on the anatomical involvement of the spinal region into two cohorts: solitary and multiple regions (multifocal) affected. Spinal regions were defined as cervical = occiput/C0–C7; thoracic = T1–T12; lumbar = L1–L5; and sacral = S1–S5 (including coccyx). Multifocal involvement was recorded when ≥2 of these regions were affected continuously or non-contiguously in the same patient.

Readmissions were tracked utilizing the unique VisitLink identifiers provided by NRD. Time to readmission was measured as the interval (in days) between initial hospital discharge and subsequent hospital admission. Mortality was restricted to deaths occurring within the index hospitalization period, thus only reflecting immediate inpatient mortality.

### 2.4. Statistical Analysis

Descriptive statistical measures included frequency and percentage calculations for categorical variables, while continuous variables were summarized using mean values and standard deviations. Differences between categorical variables were assessed using Chi-square tests, with independent samples *t*-tests used for continuous data. A multivariable logistic regression model was developed to investigate the association between spinal involvement and readmission, using solitary-region spinal involvement as the reference group. Collinearity among covariates was evaluated prior to analysis. Covariates selected for multivariable models were identified based upon significant bivariate analysis results (*p* ≤ 0.05), known risk factors, and expert recommendations. Analyses were performed using SPSS (IBM^®^ SPSS Statistics^®^, Version 29.0.2.0, Armonk, NY, USA).

## 3. Results

### 3.1. Demographics

A total of 6132 patients met the inclusion criteria, of whom 5547 (90.4%) had solitary spondylodiscitis and 585 (9.6%) had multifocal involvement. Their demographic characteristics are summarized in Table 1.

We found that patients with multifocal disease were slightly younger (solitary 60.29 years ± 15.91 vs. multifocal 58.94 years ± 14.76; *p* = 0.049) and had longer hospital stays (11.40 days ± 14.21 vs. 14.70 ± 14.50 days; *p* < 0.001). The Elixhauser indices, reflecting comorbidity burden, differed significantly between the groups. The in-hospital mortality index was lower in the multifocal group (solitary −0.45 ± 9.27 vs. multifocal −1.38 ± 9.15 vs.; *p* = 0.021), while the Elixhauser 30-day readmission index was higher (5.44 ± 5.61 vs. 6.03 ± 5.86; *p* = 0.016). Among various comorbidities, only patients with diabetes mellitus type 2 differed significantly (solitary 28.40 vs. multifocal 32.99%; *p* = 0.020). Although frequent, hypertension (solitary 63.03 vs. multifocal 65.13%) did not differ among the groups. All the other comorbidities included in the analysis also did not differ significantly between the cohorts. In-hospital mortality was excluded from the analysis because the incidence was below the Healthcare Utilization Project reporting minimum due to privacy protection guidelines.

Among the 585 patients suffering from multifocal spondylodiscitis, the majority involved two spinal regions (519 patients, 88.7%). Only 66 patients (11.3%) had three or more regions affected (Table 2).

### 3.2. Outcomes

The readmission rates within 90 days were similar and did not differ significantly between the study groups. The results are summarized in Table 3. Patients suffering from a solitary spondylodiscitis had a readmission rate of 34.9% compared to 35.6% in those with a multifocal disease (*p* = 0.766). However, time to readmission was marginally longer in the multifocal cohort (37.14 ± 24.28 vs. 35.60 ± 22.85 days; *p* < 0.001). Surgical intervention during the index admission was significantly more commonly utilized for patients with multifocal disease (25.1% vs. 20.0%; *p* = 0.003).

### 3.3. Multivariable Analysis Evaluating the Effect on the Readmission Rate

The multivariate logistic regression (C-statistic = 0.55; Hosmer–Lemeshow χ^2^ = 27.6, df = 8, *p* = 0.0006; McFadden R^2^ = 0.008), using the solitary disease cohort as reference, revealed several findings: surgery at index admission and initial length of stay were both found to be protective of readmissions (Table 4). Surgery was associated with a 26% reduction in odds of 90-day readmission (aOR 0.743, 95% CI 0.646–0.854; *p* < 0.001), and each additional hospital day decreased readmission risk by 1% (aOR 0.990 per day, 95% CI 0.986–0.994; *p* < 0.001). Diabetes mellitus type 2 emerged as the only independent risk factor for readmission (aOR 1.236, 95% CI 1.099–1.390; *p* < 0.001). In contrast, multifocal spondylodiscitis had no discernible impact on readmission odds. Neither involvement of two regions (aOR 1.121, 95% CI 0.929–1.353; *p* = 0.236) nor disease manifestations in three or more regions (aOR 0.688, 95% CI 0.394–1.203; *p* = 0.189) significantly influenced readmission risk compared to solitary disease patients.

## 4. Discussion

Spondylodiscitis continues to be an increasing burden on healthcare systems worldwide, with its adverse implications on costs of care and associated risk of morbidity and mortality despite improvements in diagnostic tools [4,18,19,20,21,22]. While there is a growing body of knowledge for spondylodiscitis in general, specifically for multifocal, there are relatively few dedicated studies available to date. Given the globally increasing incidence of spondylodiscitis, an enhanced understanding of risk factors that affect patient outcomes is desirable [5].

In our database investigation we found 9.6% of patients to suffer from a multifocal spondylodiscitis. This confirms the incidences reported by smaller, single-center studies ranging from 13% up to 35% [11,12,19]. The differences in rates may mainly be elucidated by the differences in cohort size. Compared to the single-center studies, our cohorts were more >60-fold larger. Thus, our estimation of the incidence of a multifocal disease may be more accurate.

In our study, the patients who suffered from multifocal disease were slightly younger, with solitary patients being on average 60.29 ± 15.91 years old and multifocal patients being on average 58.94 ± 14.76 years old. Additionally, the Elixhauser comorbidity indices were different between the groups, with in-hospital mortality being lower in the multifocal group, though with a higher 90-day readmission rate. This is in contrast to a previous single-institution study of 79 patients comparing solitary and multifocal infections, which found no differences in comorbidities by the Charlson comorbidity index as well as no difference in age between the groups [14]. However, the patients with multifocal disease in our population predictably had longer hospital stays given increased infection burden, consistent with findings of the previous study. The observed differences may also be influenced by differences in sample size, comorbidity indices used, and weighting within those. The difference in age may be elucidated by several factors. Thavarajasingam et al. reported a 44% rise in spondylodiscitis admissions between 2012 and 2021, with an increase of 91% occurring in the 60–64-year group, 117% in those aged 70–74, and 133% aged 75–59. Overall, these results indicate that spondylodiscitis is no longer confined to the very old but increasingly affects younger working-age adults. This observation may be driven by higher rates of intravenous drug use, biologic immunosuppression, and S. aureus bacteremia [14].

Diabetes is a known risk factor for the development of spondylodiscitis, resulting in defective cytokine signaling, vascular changes, and increased risk of skin and urinary tract infections, which can spread hematogenously [22]. In our study, type II diabetes mellitus was the only comorbidity that significantly differed between our two study cohorts (*p* = 0.020) and was an independent risk factor for readmission (OR 1.236, CI 1.099–1.390, *p* ≤ 0.001. This is in contrast to a small previous single-center study, which did not identify a difference in risk factors comparing patients with solitary and multifocal disease aside from an increased risk of multifocal infection when the cervical/thoracic region was affected [13].

Other comorbidities such as obesity, hypertension, depression, autoimmune disease, chronic lung disease, thyroid disease, and renal failure did not differ between the groups.

Readmission rates did not significantly differ between patients with solitary and multifocal disease (34.9% vs. 35.6%, respectively; *p* = 0.766). We also found no independent association between multifocality—including cases involving three or more non-contiguous levels—and 90-day all-cause readmission. However, the patients with multifocal disease had a significantly longer time interval to readmission (37.14 ± 24.28 vs. 35.60 ± 22.85 days; *p* < 0.001). Although these results may feel counterintuitive, taken together with our findings that patients with multifocal disease had longer hospital stays and underwent surgery more frequently during their index admission, it is not surprising that patients with solitary infections had a shorter time to readmission given their decreased likelihood of receiving definitive primary surgical care during their primary stay and failed conservative management then subsequently prompting patients to return to the hospital again for further care. This is supported by our multivariate analysis as both surgery at index of admission and a longer length of stay were found to be protective against readmission (aOR = 0.743 and 0.990, respectively, and *p* < 0.001) and is in line with previous studies that have outlined early surgical intervention, including introduced minimal invasive techniques, as superior to conservative management in the treatment of spondylodiscitis [23,24,25,26]. However, LOS may act as a surrogate for measured and unmeasured factors—such as surgical treatment, completion of intravenous-to-oral antibiotic transition, optimization of comorbidities, social work coordination for post-acute care, and a more cautious discharge threshold—rather than reflecting disease severity alone. By contrast, very short stays may indicate premature discharge or restricted diagnostic work-up, thereby predisposing to early return.

Another factor, aside from differences in baseline characteristics, that may partially elucidate our findings is a “risk–treatment paradox”: clinicians may perceive multifocal infection as inherently high risk and consequently pursue earlier imaging, escalated empirical antibiotics, and a lower threshold for surgical treatment, thus influencing readmission rates.

The currently accepted diagnostic standard for spondylodiscitis diagnostics places an emphasis on imaging the entire spinal column to screen for non-contiguous infectious manifestations at other spinal regions [13,14,15,21]. In our study we indeed found that 9.6% of patients had multifocal spondylodiscitis, who in turn received treatments different from patients with solitary disease, but were not affected by different readmission rates. Our findings do underscore the benefits of performing neural imaging of the entire spinal axis to screen for multifocal spondylodiscitis.

A recent publication by Balcescu et al. further supported an increase in multifocal infections of patients with de novo primary pyogenic spondylitis [13]. In this context of a potential increase in the incidence of multifocal spondylodiscitis, the effects of pathogens may be worthy of further investigation, as primary pathogens can remain unidentified in up to approximately 40% of cases [27,28,29]. In addition, presence of multiple pathogens may be a further confounding factor [14]. Overall, treatment and diagnostic algorithms still remain unstandardized across healthcare systems. While the majority of patients with spondylodiscitis can be managed with non-surgical care, surgery is usually recommended for more severe cases, including patients with neurological compromise, progressive spinal deformity/instability, and failed medical management. Especially, treatment decisions seem to be more anecdotally and institutionally based rather than using novel evidence-based algorithms. Thus, decision-making tools such as the recently introduced SITE-Score were developed. However, these scores do not include a unique multifocal variable.

Including a multifocal determinant into novel diagnostic or treatment algorithms like the SITE-Score may be beneficial [6,7,8]. Ultimately, these considerations underscore that our understanding of spondylodiscitis, especially multifocal, is still evolving, and our management strategies are multifactorial, with early detection of disease spread and impact being a key tenet of care determination.

While our study helped identify important risk factors and evaluate some care outcomes differentiating solitary versus multifocal spondylodiscitis that are not currently reflected in the literature, a distinct limitation of our study is a lack of pathogen identification, as information regarding the causative pathogen was not sufficiently indexed in the database used. Further limitations inherent to database studies include a lack of an ultimate understanding of the severity of infectious disease burden, as well as details such as the type of antibiotic used and length of treatment applied, use of adjuvant modalities like bed rest or bracing, and any specific information regarding surgical management.

Furthermore, our database was extracted during the COVID-19 pandemic, when hospital protocols involving admission and treatment were inconsistent with non-pandemic times. Lastly, type I errors are a possible consequence of database studies, which may reveal associations that are not clinically meaningful. Future prospective studies may elucidate the findings of our analysis.

## 5. Conclusions

SD still remains a diagnostic and therapeutic challenge with limited resources available regarding multifocal spondylodiscitis disease. In our study we found a relevant portion of patients (9.6%) to suffer from multifocal SD. However, we found no significant difference in all-cause 90-day readmission rates. Despite multifocal involvement not being independently associated with readmission, surgical treatment emerged as a protective factor for both cohorts—solitary and multifocal patients—while diabetes mellitus type 2 was identified as a risk factor. Further studies investigating multifocal SD would be beneficial.

## Figures and Tables

**Table 1:** Demographics and comorbidities.

	Solitary N = 5547	Multifocal N = 585	*p*-Value
**Demographics**	**N (%) or**	**Mean (±SD)**	
**Age**	**60.29 ± 15.91**	**58.94 ± 14.76**	**0.049**
Male	3324 (59.9%)	367 (62.7%)	0.187
**Length of stay (days)**	**11.40 ± 14.21**	**14.70 ± 14.50**	**<0.001**
Non-elective admission	5198 (93.7%)	559 (95.6%)	0.076
**Elixhauser in-hospital mortality index**	**–0.45 ± 9.27**	**–1.38 ± 9.15**	**0.021**
Elixhauser 30-day readmission index	5.44 ± 5.61	6.03 ± 5.86	0.016
**Comorbidities**			
Obesity	869 (15.67)	100 (17.1)	0.368
**Diabetes mellitus type 2**	**1575 (28.4)**	**193 (32.99)**	**0.020**
Hypertension	3496 (63.03)	381(65.13)	0.316
Depression	786 (14.17)	91 (15.56)	0.363
Autoimmune disease	227 (4.09)	31 (5.3)	0.167
Chronic lung disease	1006 (18.14)	106 (18.12)	0.992
Thyroid disease	911(16.72)	88 (15.04)	0.390
Heart failure	768 (13.85)	81 (13.85)	1.000
Renal failure	379 (6.83)	41 (7.01)	0.873

Bold variables indicate statistical significance.

**Table 2:** Multifocal infections.

	Solitary N = 5547	Multifocal N = 585
**Regions of the Spine Affected**	**N (%)**	
1	5547 (100)	0 (0%)
2	-	519 (88.7%)
3 or more	-	66 (11.3%)

**Table 3:** Outcomes.

	Solitary N = 5547	Multifocal N = 585	*p*-Value
**Outcomes**	**N (%) or**	**Mean (±SD)**	
Readmission	1938 (34.9%)	208 (35.6%)	0.766
**Time to readmission**	**35.60** **± 22.85**	**37.14** **± 24.28**	**<0.001**
**Surgery at index admission**	**1109 (20)**	**147 (25.1)**	**0.003**

Bold variables indicate statistical significance

**Table 4:** Multivariate regression analysis to estimate effects on readmission using the solitary group as reference.

	Odds Ratio	95% Confidence Intervals	*p*-Value
Age	0.997	0.993–1.001	0.076
**Surgery at index admission**	**0.743**	**0.646–0.854**	**<0.001**
**Length of stay**	**0.990**	**0.986–0.994**	**<0.001**
Two regions affected	1.121	0.929–1.353	0.236
Three or more regions affected	0.688	0.394–1.203	0.189
**Diabetes mellitus type 2**	**1.236**	**1.099–1.390**	**<0.001**

Bold variables indicate statistical significance.

## Data Availability

The dataset is publicly available from the Healthcare Cost and Utilization Project (HCUP), Agency for Healthcare Research and Quality.

## Abbreviations

The following abbreviations are used in this manuscript:

SD	Spondylodiscitis
ICD-10	International Classification of Diseases, 10th Revision
NRD	Nationwide Readmissions Database
HCUP	Healthcare Cost and Utilization Project
CCSR	Clinical Classifications Software Refined
LOS	Length of Stay
aOR	Adjusted Odds Ratio
CI	Confidence Interval
COVID-19	Coronavirus Disease 2019
